# Adjuvant chemotherapy versus observation after radical cystectomy in patients with node-positive bladder cancer

**DOI:** 10.1038/s41598-019-44504-9

**Published:** 2019-06-05

**Authors:** Sahyun Pak, Dalsan You, In Gab Jeong, Cheryn Song, Jae-Lyun Lee, Bumsik Hong, Jun Hyuk Hong, Choung-Soo Kim, Hanjong Ahn

**Affiliations:** 1 Department of Urology, Center for Urologic Cancer, National Cancer Center, Goyang, Korea; 2 Department of Urology, Asan Medical Center, University of Ulsan College of Medicine, Seoul, Korea; 3 Department of Oncology, Asan Medical Center, University of Ulsan College of Medicine, Seoul, Korea

**Keywords:** Bladder cancer, Surgical oncology, Chemotherapy

## Abstract

This retrospective study compared adjuvant chemotherapy (AC) versus observation after radical cystectomy (RC) in patients with node-positive bladder cancer (pN+). Outcomes were reviewed in patients with pTanyN1-3M0 bladder cancer who underwent RC with or without AC between 1995 and 2017. Baseline characteristics between the two groups were controlled with inverse probability of treatment weighting (IPTW)-adjusted analyses. Of 281 enrolled patients, the 3-year IPTW-adjusted rates of overall survival was higher in the AC group than the RC group (46.4% vs. 33.7%, p = 0.024). AC was an independent predictor of overall survival (hazard ratio = 0.48; P < 0.0001). When patients were subdivided by lymph node density (LND), the 3-year overall survival rates were similar between the AC and RC groups in patients with LND < 9%, but higher in the AC group in patients with LND 9–25% (53.4% vs. 23.7%) and LND ≥ 25% (27.4% vs. 16.1%). The numbers needed to treat to prevent one death at 3 years were three and nine in patients with LND 9–25% and ≥25%, respectively. In conclusion, AC after RC was associated with improved overall survival in patients with node-positive bladder cancer. Patients with an intermediate nodal burden may benefit most from AC.

## Introduction

Although neoadjuvant chemotherapy prior to surgery was established as the standard of care in advanced bladder cancer based on level I evidence^1^, the role of adjuvant chemotherapy (AC) followed by surgery has not yet been fully determined^2^. Although randomized trials have been conducted on the benefit of AC in advanced bladder cancer from the early 1990s^3–6^ until recently^7–10^, these studies evaluated chemotherapy regimens that are no longer in use or had methodological flaws.

Furthermore, it is not known whether the effectiveness of AC in lymph node-positive (pN+) bladder cancer is associated with any clinicopathologic features. pN+ disease was found in approximately 20–25% of patients with muscle-invasive bladder cancer who underwent radical cystectomy (RC) with lymph node dissection^11^. Several studies to date have investigated the efficacy of AC in patients with pN+ bladder cancer, with mixed results^2,10,12–16^.

This study aimed to evaluate the impact of AC after surgery on survival outcomes in patients with pN+ bladder cancer. We also attempted to identify patients with pN+ bladder cancer that would be most likely to benefit from AC after RC.

## Materials and Methods

### Study population and outcome

The study protocol was approved by the Institutional Review Board of Asan Medical Center (no. 2018-0591). The need for informed consent was waived by the institutional review board due to the minimal risk retrospective study. The study design followed all relevant principles of the Declaration of Helsinki. Medical records of consecutive patients who underwent radical cystectomy with lymph node dissection and were diagnosed with pTanyN1-3M0 bladder cancer at Asan Medical Center between January 1998 and December 2017 were reviewed. Patients who had received radiotherapy, were pathological M1, did not undergo removal of ≥10 lymph nodes, or had inadequate clinical data were excluded. After exclusion, 281 patients were enrolled. Patient data, including clinicopathologic features, demographic characteristics, preoperative and postoperative variables, and survival outcomes, were evaluated retrospectively.

Patients were subdivided into two groups based on postoperative management. The RC group consisted of patients who underwent RC alone without adjuvant treatment, and the AC group consisted of patients who underwent AC after RC. The perioperative treatments and chemotherapeutic regimens were not randomized and were determined based on clinician or patient preferences. In the AC group, 159 patients were treated with cisplatin-based chemotherapeutic regimens, with a median of four cycles; of these, 134 (84.3%) patients completed a median of four cycles of gemcitabine and cisplatin, and 25 (15.7%) received a median of four cycles of methotrexate, vinblastine, doxorubicin, and cisplatin. All enrolled patients underwent lymph node dissection including the obturator, internal iliac, external iliac, presacral, and common iliac lymph nodes. The main outcome was overall mortality. Tumors were staged according to the seventh American Joint Committee on Cancer (AJCC) 2010 TNM staging system.

### Statistical analysis

Differences in baseline characteristics between the two groups were controlled with inverse probability of treatment weighting (IPTW)-adjusted analyses. The propensity score was estimated with surgery alone as the dependent variable by multiple logistic regression analysis. A full non-parsimonious model was developed that included all the variables in Table 1 and the interaction terms between variables. Model discrimination was assessed with c statistics (=0.756), and model calibration was assessed with Hosmer-Lemeshow statistics [Chi-square = 6.221, degrees of freedom (DF) = 8; p = 0.623]. The absolute standardized differences were used to diagnose the balance. All absolute standardized differences after weighting were less than 0.1. To avoid immortal-time bias, the landmark analysis method was used with 4 months after surgery as the fixed time. To evaluate the effectiveness of AC, the number needed to treat, defined as the average number of patients who must be treated to prevent one detrimental outcome, was calculated.

**Table 1:** Baseline characteristics of the patients.

	Unweighted population			Weighted population*		
	Surgery alone N = 122	Adjuvant chemotherapy N = 159	Standardized differences (%)	Surgery alone	Adjuvant chemotherapy	Standardized differences (%)
Age, yr	66.3	61.2	−53.1	62.6	62.5	−0.6
Gender			16.7			−8.1
Male	102 (83.6)	142 (89.3)		67.4 (85.3)	126.0 (88.1)	
Female	20 (16.4)	17 (10.7)		11.6 (14.7)	17.0 (11.9)	
Charlson comorbidity index			−37.5			7.9
0 or 1	95 (77.9)	145 (91.2)		68.0 (86.1)	126.8 (88.7)	
≥2	27 (22.1)	14 (8.8)		11.0 (13.9)	16.2 (11.3)	
Neoadjuvant chemotherapy	32 (26.2)	18 (11.3)	−38.9	12.5 (15.8)	22.6 (15.8)	−1.9
Pathologic T			−6.2			3.9
≤T2	13 (10.7)	14 (8.8)		8.9 (11.3)	14.4 (10.1)	
T3–4	109 (89.3)	145 (91.2)		70.1 (88.7)	128.6 (89.9)	
Pathologic N			5.2			5.3
N1	36 (29.5)	44 (27.7)		22.9 (29.0)	42.5 (29.7)	
N2	50 (41.0)	69 (43.4)		29.8 (37.7)	56.4 (39.4)	
N3	36 (29.5)	46 (28.9)		26.3 (33.3)	44.1 (30.9)	
Total LN removed	24.4	27.0	15.9	25.3	24.1	−7.9
Positive LN	4.6	6.4	22.8	5.4	4.8	−9.9
CIS	55 (45.1)	50 (31.4)	−28.3	29.2 (37.0)	49.3 (34.5)	−5.2
LVI	91 (74.6)	127 (79.9)		62.5 (79.1)	113.4 (79.3)	0.6
Positive margin	10 (8.2)	16 (10.1)	6.5	5.5 (7.0)	12.0 (8.4)	−5.1
Histology			17.9			4.3
Pure UC	79 (64.8)	114 (71.7)		56.7 (71.8)	103.8 (72.6)	
Variant UC histology	43 (35.2)	45 (28.3)		22.3 (28.2)	39.2 (27.4)	
Type of event						
Metastasis	69 (56.6)	99 (62.3)				
Cancer-specific death	74 (60.7)	94 (59.1)				
Overall death	98 (80.3)	114 (71.7)				

LN, lymph node; CIS, carcinoma *in situ*; LVI, lymphovascular invasion; UC, urothelial carcinoma.

Results are reported as n (%) unless otherwise indicated.

^*^To avoid immortal-time bias, the landmark analysis method was used with 4 months after surgery as the fixed time.

Clinicopathological data were expressed as frequencies and means. Differences in categorical variables between groups were assessed using the Chi-square test, and differences in continuous variables were assessed using Student’s t-tests. Variables prognostic for survival were assessed by multivariate analysis using the stratified time-varying covariate Cox model with a stepwise backwards elimination approach. Covariates in the final model were selected based on the statistical significance in univariate analysis (p < 0.2). Survival outcomes were determined using the Kaplan-Meier method and compared with log-rank tests. Competing risks regression was performed to test the association of predictor variables after accounting for other-cause mortality. All statistical tests were two-tailed, with a significance level of 0.05. All statistical analyses were performed using SPSS version 25.0 (IBM, Armonk, NY, USA) and R version 3.5.1 (R Foundation for Statistical Computing, Vienna, Austria).

## Results

Baseline characteristics are summarized in Table 1. Of the 281 patients with pN+ bladder cancer enrolled in this retrospective study, 122 (43.4%) underwent RC alone and 159 (56.6%) received AC after RC. The median follow-up duration for enrolled patients was 38.4 months. Age, gender, and cystectomy pathology, including pathologic stage and nodal status, did not differ significantly between the RC and AC groups. However, patients in the RC group were older and had a higher rate of receiving neoadjuvant chemotherapy before RC.

**Figure 1:**
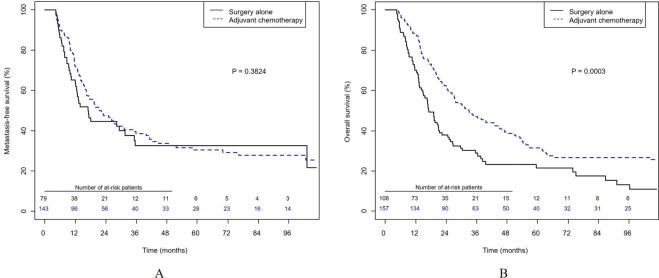
Kaplan-Meier analysis of survival in patients with node-positive bladder cancer. (**A**) Metastasis-free survival. (**B**) Overall survival.

In the unweighted study population, the 3-year metastasis-free survival rate was similar between the AC and RC groups (38.7% vs. 32.8%, p = 0.382; Fig. 1A), but patients in the AC group had a significantly higher 3-year overall survival rate than those in the RC group (47.1% vs. 28.9%, p < 0.001; Fig. 1B).

**Figure 2:**
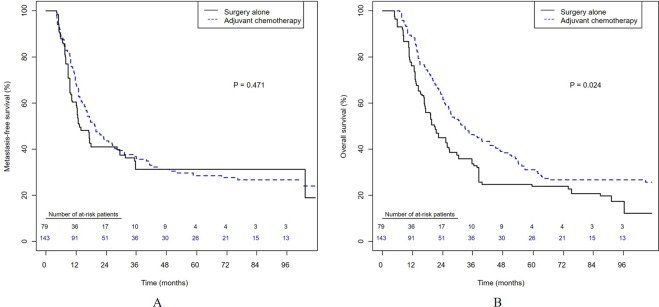
Inverse probability of treatment weighting (IPTW)-adjusted Kaplan-Meier analysis of survival in patients with node-positive bladder cancer. (**A**) Metastasis-free survival. (**B**) Overall survival.

Consistently, the 3-year IPTW-adjusted rates of metastasis-free survival were similar between the AC and RC groups (35.8% vs. 31.2%, p = 0.471; Fig. 2A), but the IPTW-adjusted overall survival rate was higher in the AC than the RC group (46.4% vs. 33.7%, p = 0.024; Fig. 2B). AC was associated with overall survival in both the time-varying Cox model (HR = 0.478; 95% CI, 0.344–0.665; P < 0.0001; Table 2) and the IPTW-adjusted model (hazard ratio (HR) = 0.663; 95% confidence interval (CI), 0.464–0.947, p = 0.024; Table 3). Competing risks regression analysis (Supplementary Table 1) showed that AC was independently associated with a decreased risk of cancer-specific death (HR = 0.641; 95% CI, 0.454–0.906; p = 0.0117).

**Table 2:** Multivariable analysis for evaluating the risk of overall mortality.

	Univariate			Multivariate*			
	Hazard ratio	95% CI	P value	Hazard ratio	95% CI		P value
**Adjuvant chemotherapy**	**0.545**	**0.411–0.722**	**<0.0001**	**0.478**	**0.344–0.665**		**<0.0001**
Age	1.028	1.013–1.044	0.0002	1.016	1.001–1.032		0.0412
Pathologic T							
≤T2	1 [Reference]				1 [Reference]		
T3–4	2.197	1.276–3.782	0.0045	2.355	1.377–4.026		0.0018
Pathologic N							
N1	1 [Reference]				1 [Reference]		
N2	1.602	1.144–2.242	0.006	1.474	1.001–2.171		0.0496
N3	1.645	1.142–2.370	0.0075	1.129	0.729–1.749		0.5861
Positive lymph nodes	1.014	1.001–1.027	0.0378	0.987	0.969–1.006		0.178
Lymph node density	1.015	1.009–1.022	<0.0001	1.018	1.010–1.026		<0.0001
Neoadjuvant chemotherapy	1.521	1.071–2.160	0.0192	1.38	0.927–2.056		0.113
Charlson comorbidity index							
0 or 1	1 [Reference]			1 [Reference]			
≥2	1.386	0.944–2.034	0.0956	0.996	0.637	1.557	0.9862

Time-varying covariate cox model with robust standard error. *Adjusted by covariates with p < 0.2 in the univariate analysis.

**Table 3:** Effect of adjuvant chemotherapy on overall survival in patients with node-positive bladder cancer.

	Hazard ratio	95% CI	P
Unadjusted	0.545	0.411–0.722	<0.0001
IPTW-adjusted	0.663	0.464–0.947	0.024
Time-varying covariate-adjusted	0.478	0.344–0.665	<0.0001

CI, confidence interval; IPTW, inverse probability of treatment weighing.

**Figure 3:**
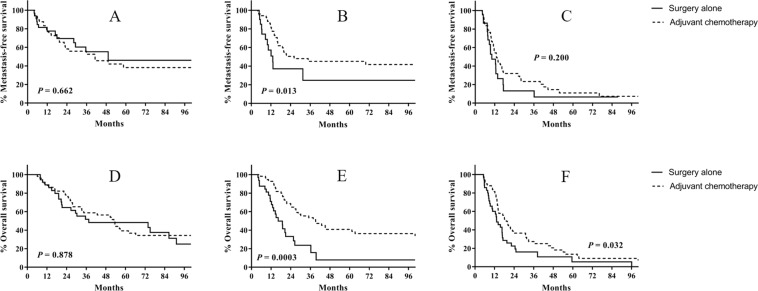
Kaplan-Meier analysis of survival in patients with node-positive bladder cancer according to lymph node density. (**A**) Metastasis-free survival in patients with lymph node density <9%. (**B**) Metastasis-free survival in patients with lymph node density 9–25%. (**C**) Metastasis-free survival in patients with lymph node density ≥25%. (**D**) Overall survival in patients with lymph node density <9%. (**E**) Overall survival in patients with lymph node density 9–25%. (**F**) Overall survival in patients with lymph node density ≥25%.

Patients were divided into three tertiles according to lymph node density (LND), as follows: <9%, 9–25%, and ≥25%. When subdivided according to LND, the 3-year metastasis-free survival rates were similar between the AC and RC groups in patients with LND < 9% (52.6% vs. 55.3%, p = 0.662) and ≥25% (23.3% vs. 13.2%, p = 0.200; Fig. 3). However, the 3-year metastasis-free survival rate was significantly higher in the AC group in patients with LND 9–25% (48.0% vs. 24.7%, p = 0.013; Fig. 3B). The 3-year overall survival rates were also similar between the AC and RC groups in patients with LND < 9% (58.7% vs. 51.7%, p = 0.878; Fig. 3D), but higher in the AC group in patients with LND 9–25% (53.4% vs. 23.7%, p = 0.003; Fig. 3E) and LND ≥ 25% (27.4% vs. 16.1%, p = 0.032; Fig. 3F). The numbers needed to treat to prevent one death at 3 years were three and nine in patients with LND 9–25% and ≥25%, respectively.

## Discussion

Despite the clear survival advantage, population-based studies show that only approximately 20% of muscle-invasive bladder cancer patients receive neoadjuvant chemotherapy, while AC is reportedly administered often, or more frequently^16,17^. Although randomized trial evidence is limited, several retrospective studies^12–16,18,19^ and meta-analyses^2,10^ have investigated whether AC is beneficial for survival in patients with muscle-invasive bladder cancer. Most studies on this issue reported a survival benefit with AC after RC in patients with muscle-invasive bladder cancer. However, the efficacy of AC in pN+ bladder cancer patients has not been fully determined. Lymph node metastasis is associated with a higher rate of metastatic progression after surgery and a lower rate of survival^11^. Some studies show that AC significantly improves the survival of patients with pN+ disease^14,16,19^, whereas others do not^10,18^.

In the present study, we reported that, in comparison with RC alone, AC after RC was associated with a survival benefit in pN+ bladder cancer patients in both an IPTW-adjusted analysis and a time-varying Cox model. The LND, the number of positive lymph nodes divided by the total number of lymph nodes removed, was introduced as a novel approach to stratify pN+ patients^20^. The LND has been reported to be a useful prognostic tool because it reflects the nodal disease burden and the meticulousness of the node dissection during RC^21,22^. Moreover, several studies reported that LND is superior to TNM nodal staging in predicting survival after RC^22–24^. Consistent with previous studies, in the present study, LND was an independent predictor of overall mortality, whereas the pathologic N-stage was not prognostic.

Considering that neoadjuvant chemotherapy may impact the outcome in patients who received AC, an IPTW-adjusted analysis was performed to correct for any neoadjuvant chemotherapy effect. In one recent study, AC administration after surgery was associated with a significant overall survival benefit in patients treated with neoadjuvant chemotherapy in a relatively large observational cohort^15^. However, in the present study, Kaplan-Meier analysis showed that overall survival was similar in neoadjuvant chemotherapy-treated patients in the AC group and the RC group (Supplementary Fig. 1). This disparate outcome may be due to the limited number of patients treated with neoadjuvant chemotherapy in the present study cohort (n = 49) and any unadjusted confounding factors. Competing risks regression analysis showed that neoadjuvant chemotherapy was associated with an increased risk of cancer-specific death. Similarly, two recent studies reported that neoadjuvant chemotherapy is associated with a worse prognosis in pathologically node-positive patients who underwent RC^19,25^.

Notably, in this study, the survival benefit from AC differed according to LND. Patients were stratified into tertiles based on the LND because there is no consensus regarding the appropriate cut-off points for LND. The present study showed that adjuvant cisplatin-based chemotherapy after RC may not be beneficial in pN+ bladder cancer with a low nodal burden. The lack of difference in survival outcomes between RC alone and AC with RC in patients with LND < 9% indicates that low nodal burden disease could be cured with RC assuming meticulous lymph node dissection, similar to previous reports^22,26,27^. By contrast, AC after RC was associated with a survival benefit in patients with LND ≥ 9%. In addition, we quantified the effectiveness of AC versus surgery alone based on the numbers needed to treat. To benefit one patient, a higher number of patients would need to be treated with AC in the LND ≥ 25% group (nine) compared to the LND 9–25% group (three). These results indicate that patients with an intermediate nodal burden may benefit most from AC after RC, whereas conventional adjuvant cisplatin-based chemotherapy may be less effective in disease with extensive nodal involvement. Since the effect of AC decreased with increasing nodal burden in this study cohort, we attempted to identify a cut-off point where the benefit from AC was not significant. AC was not associated with a survival benefit in pN+ disease with LND ≥ 35% or 40%, but remained beneficial in patients with LND ≥ 30% (Supplementary Fig. 2).

The difference in survival benefit from AC in pN+ bladder cancer according to LND highlights the importance of individual postoperative treatment strategies. The results reported in this study suggest that AC should be considered in pN+ patients with an intermediate nodal burden. By contrast, careful surveillance may be a viable treatment option in pN+ patients with a low nodal burden after RC with meticulous lymph node dissection. Patients with a high nodal disease burden have a poor prognosis even with AC after surgery. Treatment with newly emerging immune checkpoint inhibitors^28,29^ or enrollment in clinical trials should be considered to prolong the survival of these patients. Recently, molecular subtype classification of advanced bladder cancer to predict chemotherapy responses showed promising results^30,31^ and may be useful in treatment planning for pN+ bladder cancer patients.

This study had several limitations. First, this study was retrospective in nature and susceptible to selection biases. The non-randomized study design and the lack of standardized protocols for perioperative chemotherapy, lymph node dissection, and salvage therapy may have introduced biases. To overcome this, an IPTW-adjusted landmark analysis was performed, but such methodology cannot account for unknown confounding factors. Therefore, caution should be exercised in drawing conclusions from this study regarding the efficacy of AC. Secondly, although this was a relatively large cohort study regarding adjuvant chemotherapy in pN+ bladder cancer patients, the number of patients limited various subgroup analyses. Our results suggest the need for prospective randomized trials comparing RC alone and AC followed by RC in patients with pN+ bladder cancer.

## Conclusions

AC administration after RC was associated with improved overall survival in patients with pN+ bladder cancer. The survival benefit from AC after RC may differ according to LND. Patients with an intermediate nodal burden may benefit most from AC, whereas the effect of AC was not apparent in low nodal burden disease. Prospective randomized studies are needed to confirm the results of the present study.

## Supplementary information

Supplementary Appendix
