# Supplementary material for: Adjuvant chemotherapy versus observation after radical cystectomy in patients with node-positive bladder cancer
Source: Sci Rep. 2019 Jun 5;9:8305. doi: 10.1038/s41598-019-44504-9 (PMC6549178; doi:10.1038/s41598-019-44504-9)
Supplement: Supplementary file 1 — Supplementary Appendix [file 41598_2019_44504_MOESM1_ESM.docx]

**Adjuvant chemotherapy versus observation after radical cystectomy in patients with node-positive bladder cancer**

Sahyun Pak^1^, Dalsan You^2^, In Gab Jeong^2^, Cheryn Song^2^, Jae-Lyun Lee^3^, Bumsik Hong^2^, Jun Hyuk Hong^2^, Choung-Soo Kim^2^, Hanjong Ahn^2^

^1^Department of Urology, Center for Urologic Cancer, National Cancer Center, Goyang, Korea.

^2^Department of Urology, Asan Medical Center, University of Ulsan College of Medicine, Seoul, Korea

^3^Department of Oncology, Asan Medical Center, University of Ulsan College of Medicine, Seoul, Korea

**Corresponding author**: Hanjong Ahn

Department of Urology, University of Ulsan College of Medicine, Asan Medical Center,

88 Olympic-ro 43-gil, Songpa-gu, Seoul 05505, Korea

Tel: 82-2-3010-3733; Fax: 82-2-477-8928; E-mail: hjahn@amc.seoul.kr

**Supplementary Appendix**

**Table of Contents**

Supplementary Figure 1. Kaplan-Meier analysis of survival in node-positive bladder cancer patients treated with neoadjuvant chemotherapy.

Supplementary Figure 2. Kaplan-Meier analysis of survival in node-positive bladder cancer patients with a high nodal burden.

(A) Lymph node density ≥ 30%.

(B) Lymph node density ≥ 35%.

(C) Lymph node density ≥ 40%.

Supplementary Table S1. Competing risks regression model for factors associated with cancer-specific mortality in patients with node-positive bladder cancer.

Supplementary Table S2. Multivariable analyses of factors associated with overall mortality.

Supplementary Figure 1. Kaplan-Meier analysis of survival in node-positive bladder cancer patients treated with neoadjuvant chemotherapy.


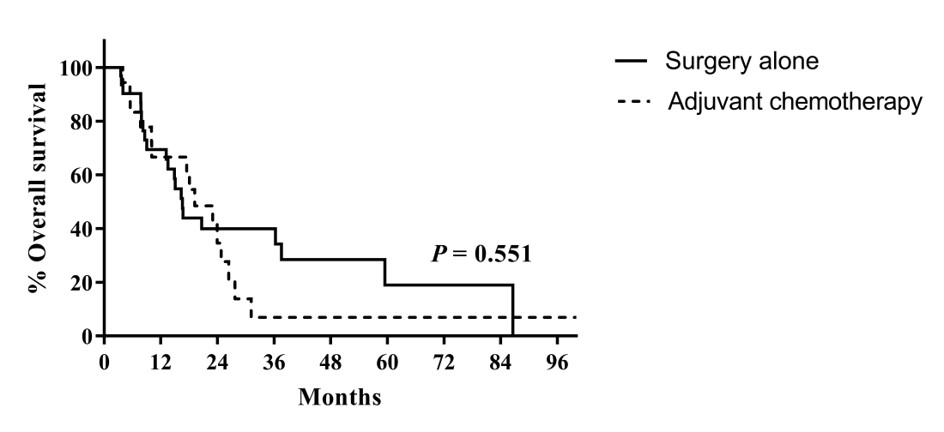


Supplementary Figure 2. Kaplan-Meier analysis of survival in node-positive bladder cancer patients with a high nodal burden.

(A) Lymph node density ≥ 30%.

(B) Lymph node density ≥ 35%.

(C) Lymph node density ≥ 40%.


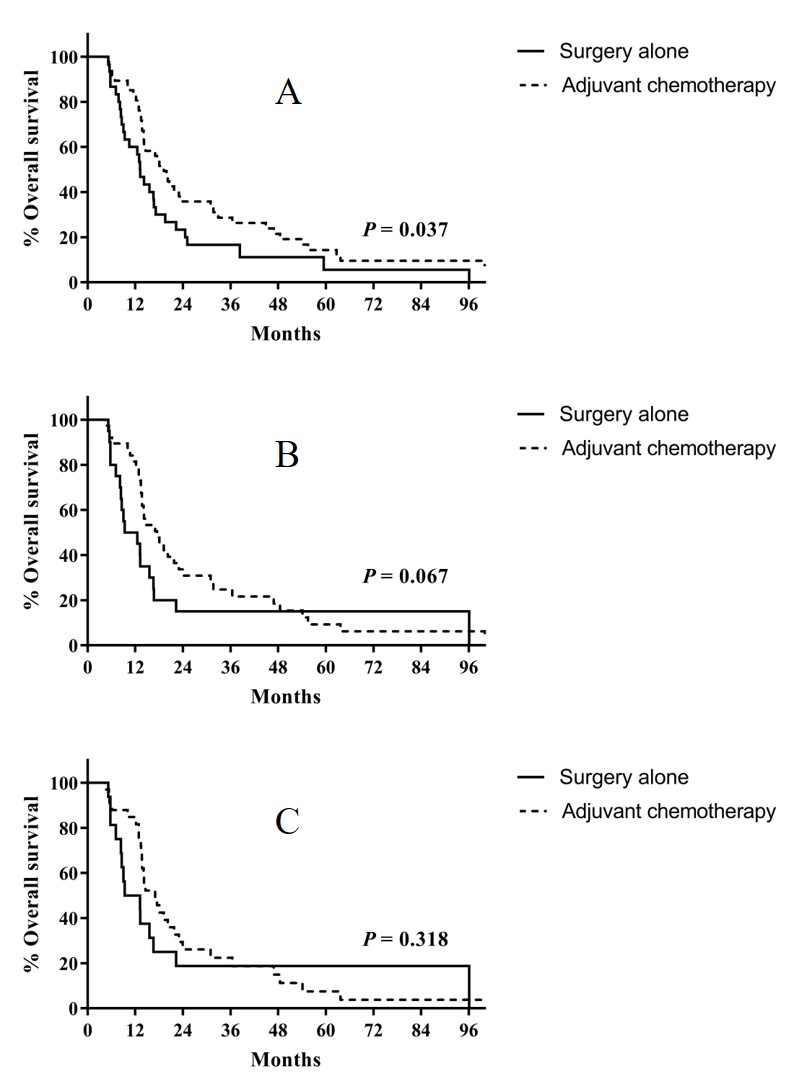


Supplementary Table S1. Competing risks regression model for factors associated with cancer-specific mortality in patients with node-positive bladder cancer.

|  |  | **Univariable** | | | |  | **Multivariable*** | | | |
| --- | --- | --- | --- | --- | --- | --- | --- | --- | --- | --- |
|  |  | HR | 95%CI |  | p value |  | HR | 95%CI |  | p value |
| **Adjuvant chemotherapy** |  | 0.718 | 0.524 | 0.983 | **0.0385** |  | 0.641 | 0.454 | 0.906 | **0.0117** |
| **Age** |  | 1.01 | 0.994 | 1.026 | 0.2389 |  |  |  |  |  |
| **Positive lymph nodes** |  | 1.01 | 0.994 | 1.027 | 0.2335 |  |  |  |  |  |
| **Lymph node density** |  | 1.015 | 1.009 | 1.022 | <.0001 |  | 1.014 | 1.007 | 1.022 | 0.0001 |
| **Pathologic T** |  |  |  |  |  |  |  |  |  |  |
| **≤ T2** |  | 1 |  |  |  |  | 1 |  |  |  |
| **T3–4** |  | 2.286 | 1.271 | 4.11 | 0.0058 |  | 2.16 | 1.158 | 4.029 | 0.0155 |
| **Pathologic N** |  |  |  |  |  |  |  |  |  |  |
| **N1** |  | 1 |  |  | 0.0015 |  | 1 |  |  | 0.0306 |
| **N2** |  | 2.045 | 1.376 | 3.041 | 0.0004 |  | 1.654 | 1.063 | 2.574 | 0.0256 |
| **N3** |  | 1.873 | 1.223 | 2.869 | 0.0039 |  | 1.133 | 0.68 | 1.887 | 0.6315 |
| **Neoadjuvant chemotherapy** |  | 1.653 | 1.131 | 2.415 | 0.0094 |  | 1.575 | 1.033 | 2.401 | 0.0348 |
| **Charlson comorbidity index** |  |  |  |  |  |  |  |  |  |  |
| **0 or 1** |  | 1 |  |  |  |  |  |  |  |  |
| **≥ 2** |  | 1.071 | 0.696 | 1.651 | 0.7542 |  |  |  |  |  |

Time-varying covariate cox model with robust standard error.

*Adjusted by covariates with p<0.2 in univariate analysis.

Supplementary Table S2. Multivariable analyses of factors associated with overall mortality.

|  |  | **Univariable** | | | | **Multivariable model 1*** | | | | **Multivariable model 2** | | | |
| --- | --- | --- | --- | --- | --- | --- | --- | --- | --- | --- | --- | --- | --- |
|  |  | HR | 95%CI |  | p value | HR | 95%CI |  | p value | HR | 95%CI |  | p value |
| **Adjuvant chemotherapy** |  | 0.545 | 0.411 | 0.722 | **<.0001** | 0.478 | 0.344 | 0.665 | **<.0001** | 0.511 | 0.375 | 0.695 | **<.0001** |
| **Age (continuous)** |  | 1.028 | 1.013 | 1.044 | 0.0002 | 1.016 | 1.001 | 1.032 | 0.0412 | 1.018 | 1.003 | 1.034 | 0.0211 |
| **Positive lymph nodes (continuous)** |  | 1.014 | 1.001 | 1.027 | 0.0378 | 0.987 | 0.969 | 1.006 | 0.178 | 0.984 | 0.965 | 1.004 | 0.1189 |
| **Lymph node density (continous)** |  | 1.015 | 1.009 | 1.022 | <.0001 | 1.018 | 1.01 | 1.026 | <.0001 | 1.02 | 1.013 | 1.027 | <.0001 |
| **Pathologic T** |  |  |  |  |  |  |  |  |  |  |  |  |  |
| **≤ T2** |  | 1 |  |  |  | 1 |  |  |  | 1 |  |  |  |
| **T3–4** |  | 2.197 | 1.276 | 3.782 | 0.0045 | 2.355 | 1.377 | 4.026 | 0.0018 | 2.407 | 1.375 | 4.212 | 0.0021 |
| **Pathologic N** |  |  |  |  |  |  |  |  |  |  |  |  |  |
| **N1** |  | 1 |  |  |  | 1 |  |  |  |  |  |  |  |
| **N2** |  | 1.602 | 1.144 | 2.242 | 0.006 | 1.474 | 1.001 | 2.171 | 0.0496 |  |  |  |  |
| **N3** |  | 1.645 | 1.142 | 2.37 | 0.0075 | 1.129 | 0.729 | 1.749 | 0.5861 |  |  |  |  |
| **Neoadjuvant chemotherapy** |  | 1.521 | 1.071 | 2.16 | 0.0192 | 1.38 | 0.927 | 2.056 | 0.113 | 1.305 | 0.896 | 1.9 | 0.1659 |
| **Charlson comorbidity index** |  |  |  |  |  |  |  |  |  |  |  |  |  |
| **0 or 1** |  | 1 |  |  |  | 1 |  |  |  | 1 |  |  |  |
| **≥ 2** |  | 1.386 | 0.944 | 2.034 | 0.0956 | 0.996 | 0.637 | 1.557 | 0.9862 | 1.134 | 0.745 | 1.725 | 0.5573 |
| **Lymphovascular invasion** |  | 0.967 | 0.72 | 1.3 | 0.826 |  |  |  |  | 0.845 | 0.616 | 1.16 | 0.2983 |
| **Carcinoma in situ** |  | 0.96 | 0.665 | 1.386 | 0.8275 |  |  |  |  | 0.899 | 0.608 | 1.328 | 0.5917 |
| **Variant histology** |  | 0.855 | 0.631 | 1.157 | 0.309 |  |  |  |  | 0.794 | 0.582 | 1.083 | 0.1455 |

* Adjusted by covariates with p<0.2 in univariate analysis.
